# Exploring the Radioprotective Indium (III) Oxide Screens for Mammography Scans Using a Three-Layer Heterogeneous Breast Phantom and MCNPX: A Comparative Study Using Clinical Findings

**DOI:** 10.3390/medicina59020327

**Published:** 2023-02-09

**Authors:** Ghada ALMisned, Wiam Elshami, Gokhan Kilic, Erkan Ilik, Elaf Rabaa, Hesham M. H. Zakaly, Antoaneta Ene, Huseyin O. Tekin

**Affiliations:** 1 Department of Physics, College of Science, Princess Nourah bint Abdulrahman University, P.O. Box 84428, Riyadh 11671, Saudi Arabia; 2 Medical Diagnostic Imaging Department, College of Health Sciences, University of Sharjah, Sharjah 27272, United Arab Emirates; 3 Department of Physics, Faculty of Science, Eskisehir Osmangazi University, Eskisehir 26040, Türkiye; 4 Institute of Physics and Technology, Ural Federal University, 620002 Ekaterinburg, Russia; 5 Physics Department, Faculty of Science, Al-Azhar University, Assiut 71524, Egypt; 6 INPOLDE Research Center, Department of Chemistry, Physics and Environment, Faculty of Sciences and Environment, Dunarea de Jos University of Galati, 47 Domneasca Street, 800008 Galati, Romania; 7 Computer Engineering Department, Faculty of Engineering and Natural Sciences, Istinye University, Istanbul 34396, Türkiye

**Keywords:** breast dosimetry, mammography, X-ray, radiation protection

## Abstract

***Background***: During mammography, a lead-acrylic protective screen is recommended to reduce radiation exposure to the unexposed breast. Objectives: This research study aimed to construct an Indium-(III)-oxide-rich tellurite-glass screen (TZI8) and compare its performance to that of lead acrylic. ***Materials and Methods***: A three-layer heterogeneous-breast phantom was developed, using the MCNPX (version 2.7.0) Monte Carlo code. An MCNPX-simulation geometry was designed and implemented, using the lead-acrylic and TZI8 shielding screens between the right and left breast. Next, the reliability of the phantom and the variations in absorption between the lead-acrylic and TZI8 glass were investigated. ***Results:*** The findings show that the TZI8-protective-glass screen offers significantly greater radioprotection than the lead-acrylic material. The quantity of total dose absorbed in the unexposed breast was much lower for TZI8 than for lead-based acrylic. The TZI8-glass screen gives about 60% more radioprotection than the lead-acrylic screen. ***Conclusion:*** Considering the toxic lead in the structure that may be hazardous to the human tissues, the TZI8-glass screen may be used in mammography examination to provide greater radioprotection than the lead-acrylic screen, in order to greatly reduce the dose to the unexposed breast.

## 1. Introduction

The diagnosis and treatment of cancer remains one of the most pressing concerns engaging scientists worldwide today [1]. This is due to the high prevalence and morbidity percentages in the latter stages, particularly in specific cancer types [2]. Although many types of cancer do not differentiate based on gender [3], it is well known that some types of cancer are more prevalent in men and some in women [4]. On the other hand, breast cancer is a kind of cancer that is often found in women [5]. Although there is no unique cause for breast cancer, genetic factors, daily habits, and several other variables might be seen as potential triggers for its growth [6] Fortunately, early detection of breast cancer allows for a high probability of controlling and effectively treating this form of cancer [7]. Many countries now include women over a particular age in breast-cancer-screening programs and actively encourage their engagement [8]. In terms of screening women through breast imaging and recognizing possible risky situations without symptoms, these screening programs are of vital importance [8]. Although breast examinations may be conducted using a variety of diagnostic techniques, including ultrasound [9], MRI [10], and mammography [11], breast-screening programs heavily rely on mammography procedures based on the use of energetic X-rays [12]. As with conventional X-ray imaging, primary X-rays arising from the source are directed onto the breast to be imaged and interact with the breast tissue, and X-rays (also referred to as secondary X-rays) whose quantity decreases after the interaction reach the image receptor, where they reach the anatomical breast tissue. The interaction of X-rays with high-density structures and adipose tissue inside the breast may differ [12]. Structures such as calcifications and solid tumors will absorb more primary X-rays, resulting in fewer X-rays reaching the image receptor to create signals for these areas [12]. Therefore, the images to be acquired for these regions would appear with more brightness in the final image. Clearly, mammography is a challenging and complex task, yet the assessment method might differ based on a variety of criteria, including breast thickness and patient age [13]. Nevertheless, this procedure is essential for early breast-cancer identification and, therefore, treatment. In contrast, the X-rays employed in mammography are ionizing electromagnetic radiation with a tendency to interact with biological tissue [14]. This interaction is a concern that must be handled with attention in terms of biological tissue, and should be implemented inside a well-documented framework. The use of X-rays in breast imaging is obligatory, given the positive situations that may be provided by their utilization, as determined by a risk–benefit analysis [15]. In terms of public health, minimizing the risks that X-rays pose to biological tissue is therefore a significant topic for researchers. During breast screening through digital mammography, each breast is routinely scanned from two unique views, namely the medio-lateral-oblique (MLO) and crania- caudal (CC) views [16]. In other words, a mammography scan exposes the patient to a total of four radiation exposures, two to each breast. In addition to dose optimization, however, optimum protection of the unexposed breast outside of the irradiation area and minimum interaction between the dose delivered to the non-target breast reduce the patient’s overall dose amount [17]. This reduction may avoid the development of existing risks and reduce the likelihood of cancer cases being triggered by radiation [18]. In this instance, it is necessary to shield the unexposed breast outside the exposure region with appropriate materials, so preventing its interaction with X-rays. Several research studies on this have been published in the literature. With the use of a lead-acrylic screen, comprised of a transparent material, Koo and Lee [17] achieved and reported significant progress in shielding the unexposed breast against radiation. According to the findings of Koo and Lee, protecting the unexposed breast other than the target breast with this lead-acrylic screen during a standard mammogram may reduce the risk of radiation-induced cancer. However, if the lead-containing substance in this transparent screen comes into touch with the breast skin, further issues may develop owing to lead’s poisonous properties [19]. For such an application, certain non-toxic, high-density glass materials with superior mechanical qualities may be recommended. In recent years, there has been an exponential growth in the usage of high-density glasses for radiation fields and scientific research in this field [20,21]. This is due to the remarkable mechanical and physical characteristics of glass materials, as well as their low manufacturing cost, non-toxicity, and structural adjustability [22,23]. In this study, our group’s high density, 8% mole indium (III) oxide-added tellurium glass [24] was used as a breast protective screen, and its protective properties on the unexposed breast were assessed throughout a broad energy range. The findings obtained for the indium (III) oxide-doped glass screen were compared to those obtained for the protective screen proposed by Koo and Lee, and the results obtained were analyzed. This study’s findings may be applicable to the further reduction in radiation exposure during mammography operations within the context of public health. In addition, there is a tendency to integrate the findings of this research with the literature on shielding-glass materials and diagnostic radiology applications. This may allow indium-(III)-oxide-reinforced tellurium shielding material to be improved to possess higher qualities and be optimized for medical purposes.

## 2. Materials and Methods

In this study, a lead-acrylic shielding screen with experimentally determined radioprotective properties was compared to an indium-(III)-oxide-doped tellurite-glass shielding screen modelled using the MCNPX Monte Carlo code. The MCNPX [25] code was validated using the standard information provided in the AAPM [26] report. This section describes the modelling process, the verification phase of the developed code, the examined dosimetry parameters, and the comparison of the lead-acrylic glass screen.

### 2.1. Definition of the Utilized Breast Model through MCNPX Code

**Figure 1:**
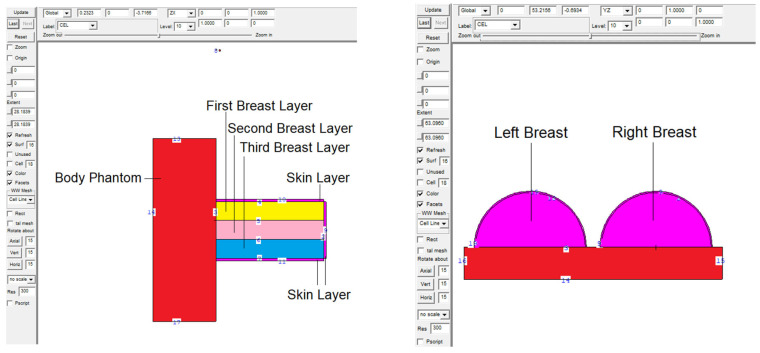
Two−dimensional view of modelled breast phantom in MCNPX code.

A body phantom was formed and positioned behind the body phantom. Recently reported in the literature, the right and left breasts were modelled as a three-layered heterogeneous breast phantom with various glandular-fraction (GF) ratios, according to the breast layers [27]. Consequently, the first, second, and third layers were divided into three-centimeters-thick cellular structures, and a geometric-design procedure was carried out for the right and left breasts. Within the volumes defined as the first, second, and third layers from top to bottom, the elemental compositions listed in Table 1 were specified as 25%, 50%, and 75% GF, respectively, in the first, second, and third layers. These volumes were assigned densities based on the elemental composition of each layer. In the data-card part of the input file, composition definition (M_n_) of the layers was carried out. Next, a model of the X-ray source was created directly above the irradiated right breast. During the second phase of the study, a protective screen was placed between the two breasts in this modelled total geometry. During the comparison phase, the shielding screen was described as lead-acrylic and indium-(III)-oxide tellurium glass (encoded as TZI8) in separate input files. Figure 1 shows lateral 2D and 3D views of the entire simulated geometry. Figure 1’s 2D and 3D images were generated using the MCNPX image editor. The 2 mm thick skin layer, whose elements composition and density are shown in Table 1, was designed to cover the right and left breasts. After the initial design phase, the modelled geometry was evaluated using X-rays with energies between 26 and 30 keV.

**Figure 2:**
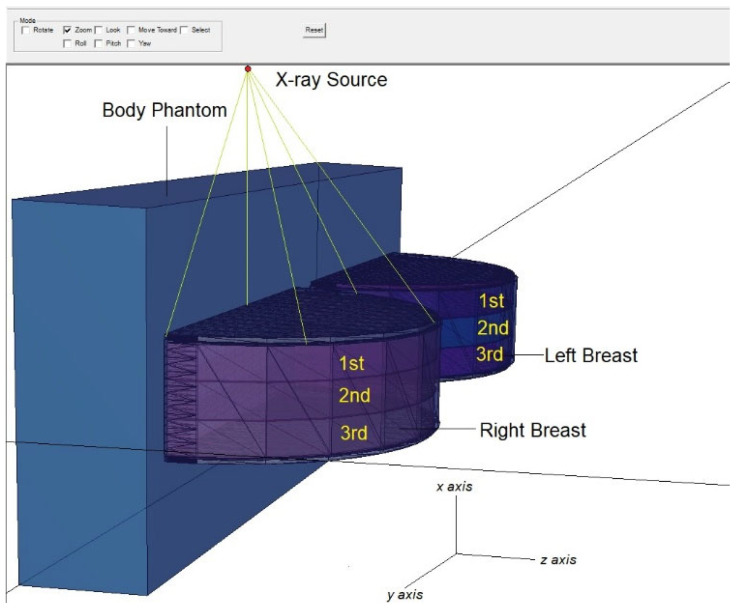
Three-dimensional view of modelled breast phantom and breast layers in MCNPX code.

**Figure 3:**
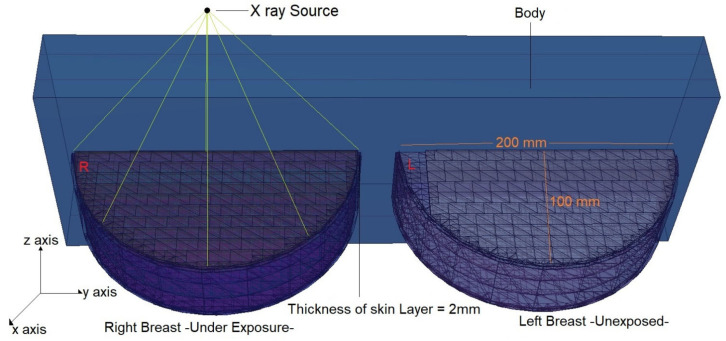
Three-dimensional view of modelled breast phantom, along with sizes and the location of X-ray source on the right breast.

Figure 2 shows the positioning of the X-ray source across the entire modelled geometry and its exposure to the right breast. Moreover, the modelled three layers [27] in the right and left breasts are seen in Figure 2. As can be seen, there is no material between the right and left breasts. For this scenario, each energy value was measured five times, and the average results were derived. During this phase, no protective equipment is used, and separate values are recorded for the right and left breasts. Figure 3 depicts the top view of the modelled overall geometry and the axial depiction of the right-breast exposure. As shown in Figure 3, the diameter of the breast model, which includes a 2 mm thick skin layer, is 200 mm and the radius is 100 mm. In the second main phase of the simulation, a protective material is defined between the right and left breasts.

**Figure 4:**
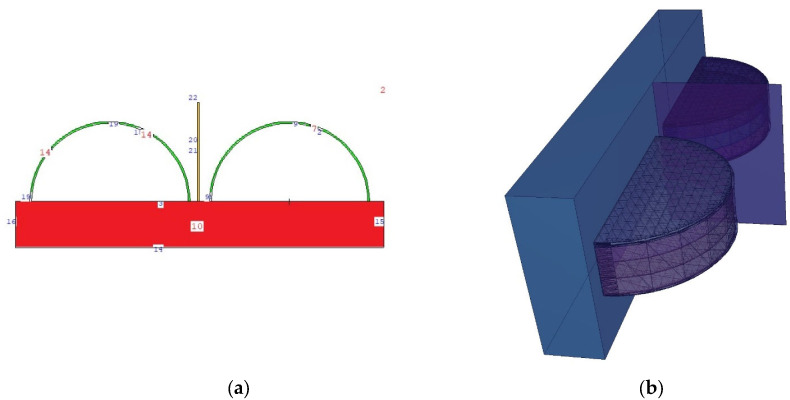
(**a**) Two-dimensional view of modelled breast phantom, along with the location of shielding screen (**b**) Three-dimensional view of modelled breast phantom, along with the location of shielding screen.

The second main simulation geometry resulting from this specification is shown in 2D (top view) and 3D in Figure 4. In the second main phase of the definition of the protective screen, the compositions of the lead-acrylic material and the TZI8 protective glass screen were defined and compared separately. Then, F6 tally meshes were defined for the skin layers and all breast layers on the right and left side. The F6 tally mesh is a tally extension for MCNPX that estimates the energy deposited in a point or volume in MeV/g. For each energy value used, the values recorded in the output file for the F6 tally meshes were recorded, and examined independently.

### 2.2. Validation of MCNPX Code

The previously published AAPM TG-195 [26] paper on dosimeter guidelines for mammographic procedures was used in the validation process of the MCNPX code. According to this approach, the breast phantom in the manual was formed utilizing the same specifications and the ratio of adipose to glandular tissue was established at 8–20%. In the modelled phantom, the energy deposition (MeV/g) was evaluated using the F6 tally-mesh extension of the MCNPX. Three cycles of counting culminated in a median value of 4798 eV/photon. Under 0.3% was the rate of observed variation. Considered a critical indicator of the dependability of the data libraries and physics lists employed in this simulation investigation, a low deviation rate was viewed as a significant indicator of its accuracy. Hence, the modelled input files were further utilized for the benchmarking phase of protective lead-acrylic and TZI8 shielding screens.

## 3. Results and Discussion

### 3.1. Initial Assessment on Transmission-Factor (TFs) Values of Lead Acrylic and TZI8

**Figure 5:**
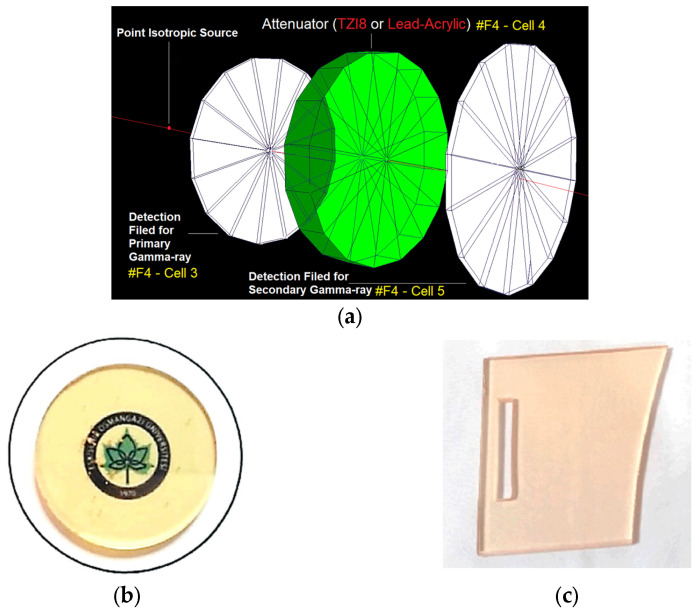
(**a**) Modelled MCNPX setup for transmission factor (TF) calculations; (**b**) physical appearance of TZI8 glass sample [24]; (**c**) physical appearance of lead-acrylic screen used by Koo and Lee [17].

Before the primary assessment step, in which the phantom and protective screens provide the changes in the amount of energy deposited in the unexposed breast, the transmission factors of the used protective screens were calculated. This computation was reviewed as a preliminary step, and the primary photon-flux reductions through the protective TZI8 and lead-acrylic shielding screens were computed and firstly compared with the following phase of calculations. The transmission factor is the ratio of the amount of secondary photon flux (also known as the secondary photon beam) passing through the absorber material to the quantity of primary photon flux (the primary photon beam) [28,29,30,31]. The small secondary-flux value is associated with the material’s high absorption, and because the primary-photon-flux value is constant, this ratio is expected to be small in materials with superior absorption properties. Figure 5 illustrates the TF-value-calculation scheme used through the MCNPX code, along with the physical appearances of the TZI8 glass sample and lead-acrylic screen.

**Figure 6:**
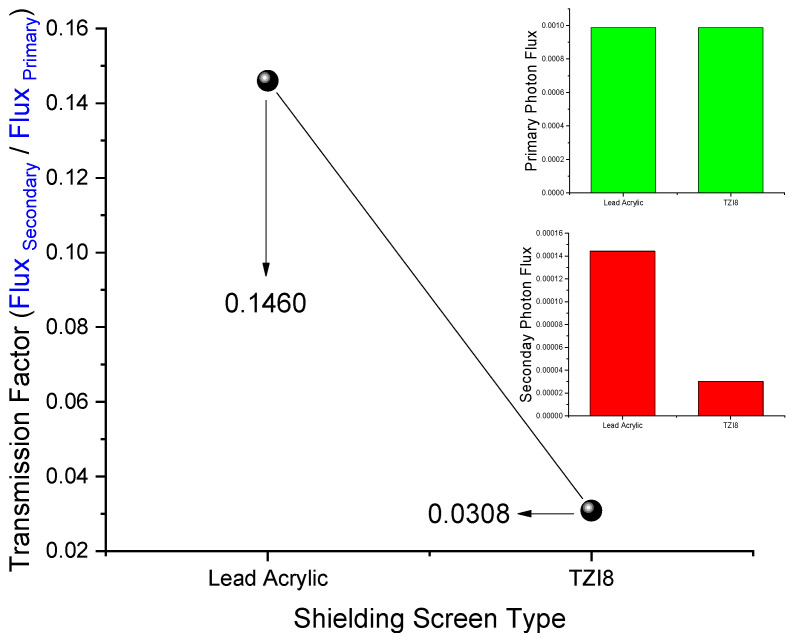
Variation of transmission factor (TF) values and comparison of primary and secondary photon-flux values.

With two F4 tally meshes defining the entrance and back side of the absorber material, the primary flux amount leaving the source (Cell 3) and the secondary flux passing the absorber material (Cell 5) were determined. The absorber material was first designated as lead acrylic and then as TZI8 on the cell card (i.e., Cell 5). For each absorber material, measurements were performed five times, and the means were determined. Figure 6 shows the variance of TF values computed using the setup shown in Figure 5. As can be seen, the primary-photon-flux values for both lead acrylic and TZI8 are similar. Since the same energy value (i.e., 0.1 MeV) and physical distances are used, this is a crucial sign for demonstrating the expected consistency of the measurements performed on both materials. However, the value obtained for TZI8 in secondary-flux amount is much smaller. This difference caused the TF values to change correspondingly, leading the 0.1460 value for lead acrylic to decline to 0.0308 levels for TZI8, and a tendency to decrease by around 80%.

### 3.2. Assesment of Lead-Acrylic and TZI8 Shielding Screens on Breast Protection

**Figure 7:**
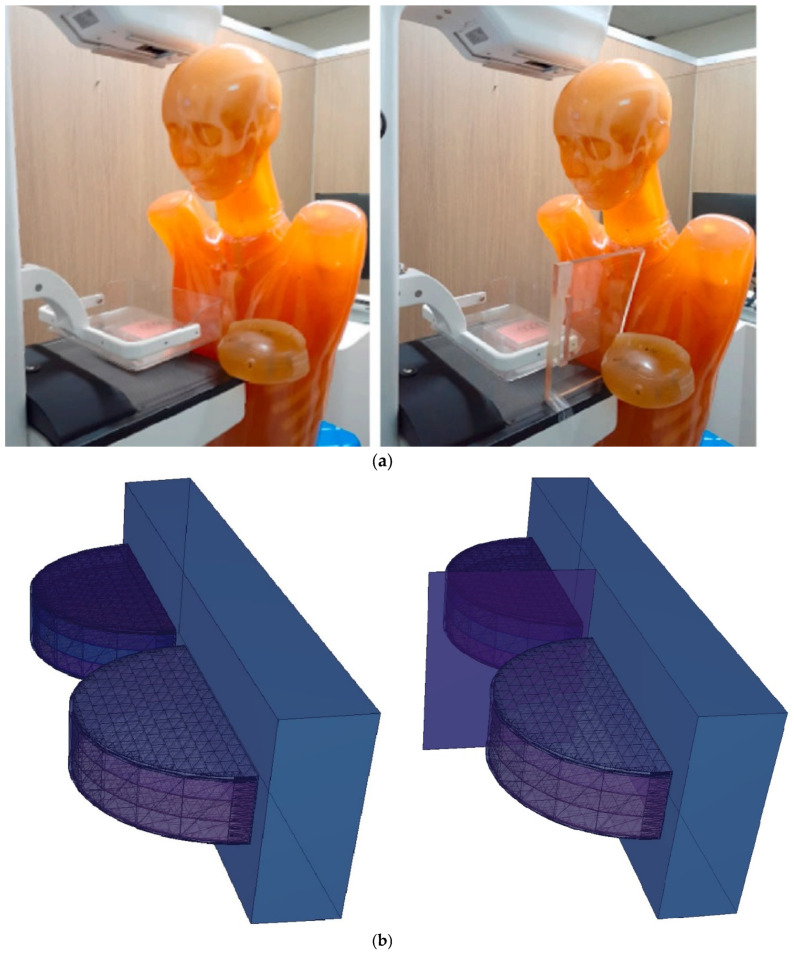
(**a**) Designed clinical setup for assessment of radio protectivity through lead-acrylic shielding screen by Koo and Lee [17]. (**b**) Designed MCNPX breast phantom using same parameters as clinical setup.

During this phase of the study, the protective efficacy of the TZI8 and lead-acrylic shielding screens on the unexposed left breast, which had been pre-assessed using the TF factor, was thoroughly investigated. In this phase investigation, the entire physical dimensions of the clinical setting shown in Figure 7a [17] were specified in the MCNPX code. The right breast was exposed without a protective screen in the first of two separate input files and with a protective screen in the second. Figure 7b illustrates the three-dimensional representation of two separate input files. During the simulation phase, the screen located between the two breasts, as seen in Figure 7b, was first designated as 12 mm thick lead acrylic, and subsequently 12 mm thick TZI8. The only difference between the two simulations is the elemental composition and density of the used screens; all other parameters remain the same.

**Figure 8:**
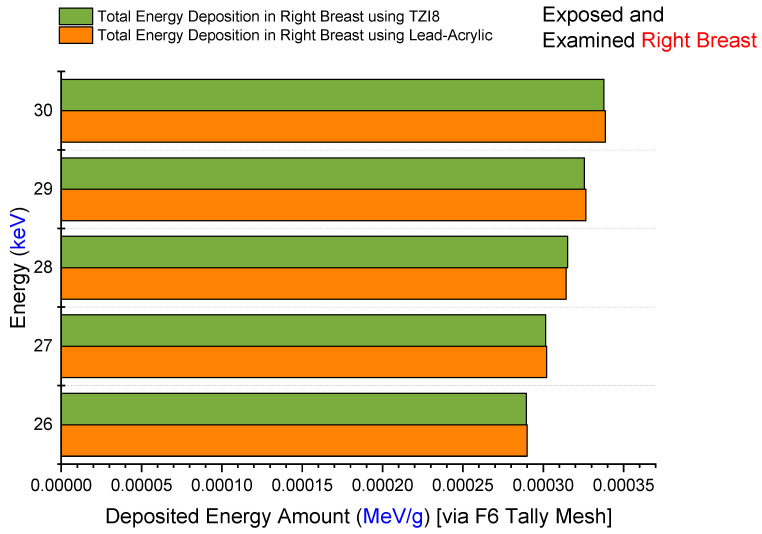
Variation in deposited-energy amount in exposed right breast.

**Figure 9:**
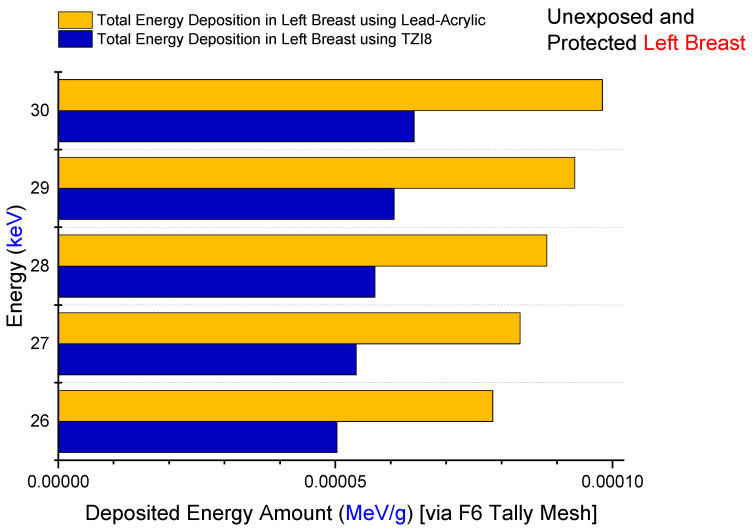
Variation in deposited-energy amount in unexposed and shielded left breast.

**Figure 10:**
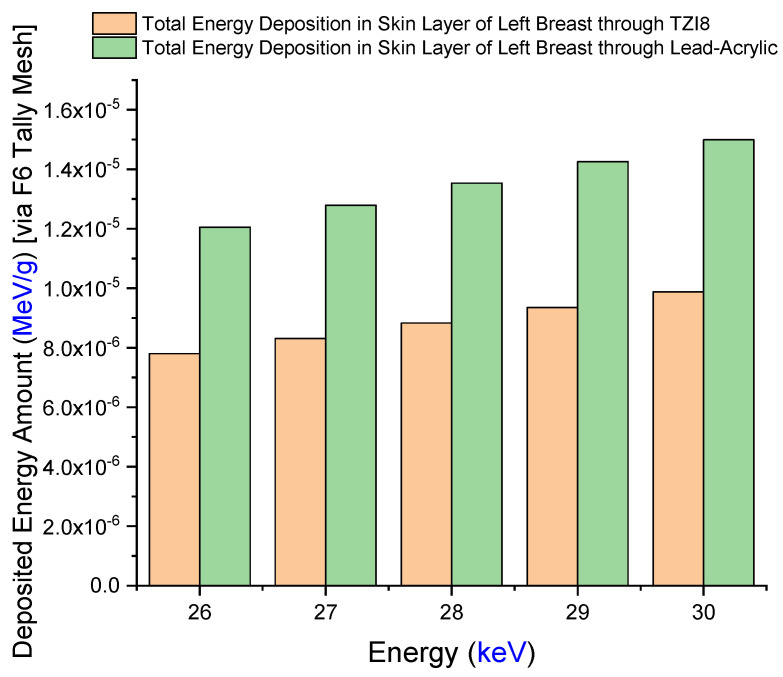
Variation in deposited energy amounts in left breast using TZI8 and lead-acrylic shielding screens.

Firstly, the amount of energy deposited in the right breast upon exposure to lead acrylic and TZI8 was determined to be between 26 and 30 keV. The quantity of energy deposited in the right breast as a function of increasing source energy is seen in Figure 8. The protective screen utilized has no influence on the exposed breast, as seen in the figure. This is a frequent phenomenon, and it demonstrates the essential X-ray exposure to the breast throughout the imaging procedure. In addition, the fact that the results derived from the exposed breast are almost same for both simulations is an essential indicator of the simulations’ quantitative consistency. As a consequence of the increase in energy from 26 keV to 30 keV, the quantity of energy deposited in the right breast increased as well. In addition to the mA value, this corresponds to the influence of the keV factor on the X-ray quantity. The doses to the left breast were then extracted from the simulation output files. Figure 9 demonstrates the fluctuation in the quantity of energy deposited in the left breast, according to utilized lead acrylic and TZI8, respectively. As shown in the figure, when TZI8 is used as a protective screen for the left breast, lower deposited-energy values for each energy value are obtained. In the direction of TZI8`s superiority, this difference is around 65% lower than the lead-acrylic screen for each energy value. With the use of TZI8, the amount of dose deposited in the left breast may be lowered by an additional 65% compared to the lead-acrylic screen. In the meantime, an increase in the quantity of energy deposited as a result of rising energy is also seen in the unexposed and protected left breast, comparable to the exposed right breast. This is a significant finding that once again underlines the importance of using the optimal dose for both exposed and protected breasts during mammography examinations. Similarly, the quantity of energy deposited in the skin layers surrounding the right/left breast, as seen in Figure 1’s lateral view, was also examined. Figure 10 depicts the total skin dose of the unexposed left breast as a function of energy for the TZI8 and lead-acrylic protective screens.

**Figure 11:**
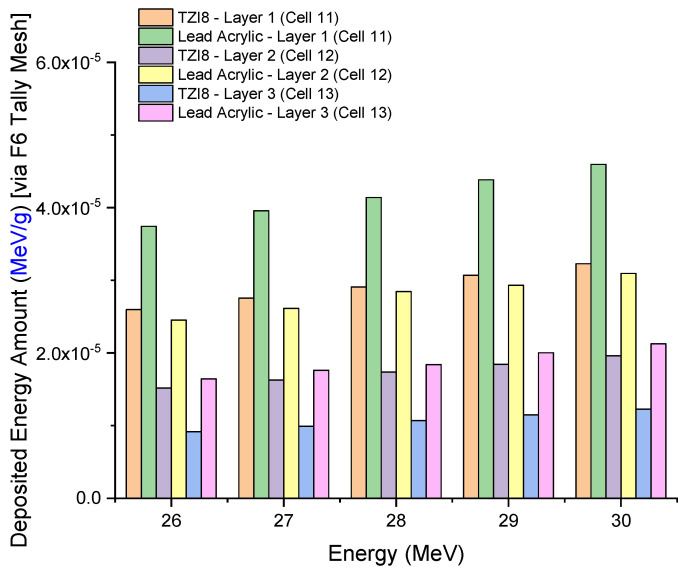
Variation of deposited-energy amounts in three breast layers of unexposed and shielded left breast, using TZI8 and lead-acrylic shielding screens.

In situations when TZI8 is implemented as a protective screen, the overall skin dose is much lower than when lead acrylic is used. In fact, this is a previously noted observation associated with total breast dose. Since the volume of the whole skin layer is much less than the geometry of the modelled breast, the values in Figure 10 are significantly less than the energy deposited in the breast. This condition may alternatively be seen as the outcome of a discrepancy in volumes. In addition, for TZI8 and lead acrylic, the amount of energy deposited in each layer of the three-layer heterogeneous breast utilized in this investigation and recently suggested in the literature was evaluated separately. Figure 11 depicts the variation of deposited-energy amounts in three breast layers of unexposed and shielded left breast, using TZI8 and lead-acrylic shielding screens. As seen in the figure, X-rays release most of their energy in the first layer, for each energy value. In addition, when TZI8 was used, the quantity of energy deposited in each layer of the preserved and unexposed left breast was less than when lead acrylic was employed. The overall quantity of energy deposited in the left breast, as described in the previous sections, is the sum of the values obtained from each layer. Consequently, the TZI8 protective screen has a clear advantage over the lead-acrylic protective screen in terms of TF values, the total amount of energy deposited in the skin layer, and the total amount of energy deposited in the protected left breast, and it is reported to be a more effective protective material for patients undergoing mammography examinations. MCNPX, one of the Monte Carlo radiation-transport codes, is confirmed using clinical data, and is one of the most widely used Monte Carlo radiation-transport methods for medical applications in the scientific literature. In addition, the fact that the generated code was highly consistent with the data shown in the AAPM TG-195 [26] reports was a significant indicator of the code’s compatibility with other extracted data. Nonetheless, this is simulation research, and it is essential to test this in clinical environments. Moreover, the obvious superiority of the outcomes based on simulation does not render these materials better in terms of cost and durability. In the next stages of manufacturing and clinical testing, the research team intends to conduct a full cost and pricing analysis, to shed some light on this limited situation.

## 4. Conclusions

The purpose of this research study was to provide a descriptive addition to the classically applied techniques, to minimize the effects of radiation from the mammography devices used in conventional breast-screening programs to the breast (left or right side) that is not directly examined. Earlier, a tellurite glass containing an 8% mole indium-(III)-oxide addition was manufactured for radiation-protection purposes, and the research team investigated various material characteristics for its use in radiation fields. Through a comparison with a lead-acrylic protection screen that has recently been recommended for breast protection during mammography examinations, our research team designed an indium-(III)-oxide tellurite-glass screen (TZI8) as a shielding material to be similar to the situations under which the lead-acrylic screen was analyzed. Based on our study’s findings, the following suggestions can be disseminated to the scientific community:

- The protection given by the TZI8-glass screen is 60–65% greater than that of the lead-acrylic screen.
- Given the drawbacks of hazardous Pb in the structure of lead-acrylic material, it can be implied that glasses rich in indium (III) oxide might be used for mammography tests, as they would provide a greater radioprotection compared to the lead acrylic, as well as significantly reducing the dose to the unexposed breast.
- Such a decrease will lower the risk of breast cancer (as a result of radiation exposure), and may be advantageous in reducing medium- and long-term concerns, by further decreasing the dose amount for the existing mammography practice.
- Given the flexibility of synthesis, the superior mechanical and thermal features of glass materials, and the transparency of TZI8 in the visible-light spectrum, it is anticipated that such a material would contribute to medical applications as a suitable breast shield.
- Using glass-shielding materials during mammography tests may be more convenient for radiographers in terms of radiographic imaging and patient positioning (owing to their transparency and, accordingly, real-time patient monitoring).

Additionally, a mammography has usually been performed yearly on patients, worldwide. In many healthcare systems, patient-centered and high-quality services are required. Consequently, protecting the breast from unnecessary harmful exposure is an opportunity to increase radiation safety in order to assure the delivery of high-quality healthcare services for the public. This was a novel investigation for maximal shielding, which is one of the procedures to be performed for patient safety in mammography examinations, which are a part of breast-screening programs. In light of the potential of glass materials for structural modification, the manufacture of this type of high-density glass in different configurations and the investigation of its protective properties will be one of the future’s top priorities. In addition, given the widespread use of mammography scans, future work will include the development of cost-effective materials with the optimal price–performance features for such materials, and where the manufacturing costs will be affordable in all countries around the world. In addition to the efficacy of medical materials, affordability in terms of price and a sustainable supply-and-demand condition are among the most important factors in the health sector. Consequently, the manufacture of materials that are maximum in terms of protection and optimum in regard to cost may be one of our group’s future research goals.

## Figures and Tables

**Table 1:** Elemental properties and densities of the modelled breast layers [27].

		Weight Percentage (%)			
Tissue	Density (g/cm^3^)	H	C	N	O
GF Tissue (25%)	0.955	11	51	2.1	35.7
GF Tissue (50%)	0.982	10.7	40.1	2.5	46.4
GF Tissue (75%)	1.010	10.5	29.3	2.9	57
Skin	1.090	9.8	17.8	5	66.7

## Data Availability

Data are available upon reasonable request.
